# Comparative genome analysis of cortactin and HS1: the significance of the F-actin binding repeat domain

**DOI:** 10.1186/1471-2164-6-15

**Published:** 2005-02-14

**Authors:** Agnes GSH van Rossum, Ellen Schuuring-Scholtes, Vera van Buuren-van Seggelen, Philip M Kluin, Ed Schuuring

**Affiliations:** 1 Department of Pathology, Leiden University Medical Center, Albinusdreef 2, 2300 RC, Leiden, The Netherlands; 2 Division of Cellular Biochemistry, The Netherlands Cancer Institute, Plesmanlaan 121, 1066 CX, Amsterdam, The Netherlands; 3 Department of Pathology, University Medical Center Groningen, Hanzeplein 1, 9700 RB, Groningen, The Netherlands

## Abstract

**Background:**

In human carcinomas, overexpression of cortactin correlates with poor prognosis. Cortactin is an F-actin-binding protein involved in cytoskeletal rearrangements and cell migration by promoting actin-related protein (Arp)2/3 mediated actin polymerization. It shares a high amino acid sequence and structural similarity to hematopoietic lineage cell-specific protein 1 (HS1) although their functions differ considerable. In this manuscript we describe the genomic organization of these two genes in a variety of species by a combination of cloning and database searches. Based on our analysis, we predict the genesis of the actin-binding repeat domain during evolution.

**Results:**

Cortactin homologues exist in sponges, worms, shrimps, insects, urochordates, fishes, amphibians, birds and mammalians, whereas HS1 exists in vertebrates only, suggesting that both genes have been derived from an ancestor cortactin gene by duplication. In agreement with this, comparative genome analysis revealed very similar exon-intron structures and sequence homologies, especially over the regions that encode the characteristic highly conserved F-actin-binding repeat domain. Cortactin splice variants affecting this F-actin-binding domain were identified not only in mammalians, but also in amphibians, fishes and birds. In mammalians, cortactin is ubiquitously expressed except in hematopoietic cells, whereas HS1 is mainly expressed in hematopoietic cells. In accordance with their distinct tissue specificity, the putative promoter region of cortactin is different from HS1.

**Conclusions:**

Comparative analysis of the genomic organization and amino acid sequences of cortactin and HS1 provides inside into their origin and evolution. Our analysis shows that both genes originated from a gene duplication event and subsequently HS1 lost two repeats, whereas cortactin gained one repeat. Our analysis genetically underscores the significance of the F-actin binding domain in cytoskeletal remodeling, which is of importance for the major role of HS1 in apoptosis and for cortactin in cell migration.

## Background

Cortactin (also designated *EMS1 *, CTTN, cttn, Amplaxin, see Genecard [1]) was initially identified as one of the most prominent tyrosine phosphorylated proteins in v-Src infected chicken embryo fibroblasts [2]. Cortactin was independently isolated from mouse NIH3T3 cells [3] and human tumor cell lines [4]. Human cortactin is encoded by the *EMS1 *gene, which is located on chromosome 11q13 [4,5]. Gene amplification of 11q13 region and concomitant overexpression of cortactin frequently occurs in several human carcinomas [4,6-8] and correlates with lymph node metastasis and increased mortality [9-11]. Elevated expression of cortactin increases cell motility, invasion [12-14] and metastasis [15].

The deduced amino acid sequence of cortactin revealed three main distinguishable domains: the N-terminal acidic domain containing a DDW-Arp2/3 binding motif followed by a six and one-half 37-amino acid F-actin binding repeat domain, a central region and an SH3 domain at the very C-terminal. The DDW-Arp2/3 binding site and the actin-binding domain together regulate F-actin polymerization and dynamics by activating the Arp2/3 complex [16] and both are necessary for translocation of cortactin to sites of actin polymerization [17]. Recently, we reported the identification of two alternative splice variants of human cortactin lacking either 6^th ^or the 5^th ^/6^th ^repeat, present in normal tissues as well as squamous cell carcinomas cell lines [14]. These splice variants differ significantly in their ability to (i) bind F-actin, (ii) cross-link F-actin (iii) activate Arp2/3 mediated actin polymerization and (iv) induce cell migration *in vitro *[14]. This indicates that also the number of repeats determines the affinity for F-actin and ability to regulate cell migration. Similar cortactin splice variants were also reported in the mouse [18], rat [19] and frog [20]. The SH3 domain is a conserved protein module found in various signal proteins and mediates the interaction with various proteins such as N-WASP involved in actin polymerization, dynamin-2 in endocytosis, ZO-1 in cell-cell interactions and SHANK-2 in neuronal growth cones (reviewed in [21]). The central part of the protein between the F-actin repeat domain and the SH3 domain contains an alpha-helix sequence and a proline-rich region with three c-Src tyrosine phosphorylation sites [22,23] and three serine/threonine phosphorylation sites [24]. Cortactin tyrosine phosphorylation occurs in response to growth factor treatment, integrin cross-linking, bacterial invasion and cell shrinkage (reviewed in [21]). Tyrosine phosphorylation of cortactin reduces its F-actin cross-linking activity and is required for its ability to stimulate cell migration [13]. Since cortactin operates mainly in cytoskeletal rearrangements, it may link other proteins via its SH3 domain to sites of actin polymerization. Alternatively, serine phosphorylation of cortactin by Erk enhances, whereas Src phosphorylation inhibits the activation of N-WASP by cortactin [25] and as a result affects actin polymerization. This suggests that cortactin at first instance may be directed to the site of actin polymerization by other proteins. Thus, changes in protein expression level, phosphorylation state, the relative expression of splice variants and interactions with other proteins can all influence cell migration.

Cortactin shows the highest similarity to the hematopoietic lineage cell-specific protein 1 (HS1). Human *HS1 *(also designated *HCLS1 *, see Genecard [26]) was originally isolated by its homology to the adenovirus E1A gene [27]. HS1 overall similarity to cortactin at the amino acid level is 51% but is highest at the SH3 domain (86%) and the 37-amino-acids repeat domain (86%), except that HS1 carries only three and one-half repeats. Despite this high homology, the function of HS1 differs considerable from cortactin. First, HS1 is mainly expressed in hematopoietic cells [27], whereas cortactin is widely expressed in all cell types except most hematopoietic cells [28]. Only in platelets and in megakaryocytes both genes are expressed [29,30]. Second, in concordance with this tissue distribution, HS1 is tyrosine phosphorylated after receptor cross-linking in B-cells [31], T-cells [32], mast cells [33] and erythroid cells [34], but at different residues compared to the functional phosphorylation residues in cortactin [13,23]. Third, HS1 is, like cortactin, a cytoplasmic protein, but after tyrosine phosphorylation HS1 translocates to the nucleus [35], whereas cortactin is never found in the nucleus. This is because HS1, but not cortactin, contains a nuclear localization signal (NLS) [36,37]. Fourth, HS1 plays an important role in the receptor-mediated apoptosis and proliferative responses as demonstrated by the analysis of HS1 deficient mice [38] and WEH1-231 B lymphoma cells [37,39]. An HS1 tyrosine mutant that could not translocate to the nucleus, also failed to induce apoptosis [37]. Consistent with its role in apoptosis, HS1 is able to bind to the mitochondrial protein HAX-1, a Bcl2 like protein [40]. Finally, the SH3 domain of HS1 at the C-terminus binds to other proteins (Ste20 related kinase HPK1 [41] and HS1-BP3 [42]) than those binding to cortactin, despite the very high amino acid sequence similarity of both SH3 domains (86%). This most probably reflects the different tissue-specific expression pattern.

Cortactin and HS1 share also remarkable similarities. First, HS1 binds with its DDW-motif directly to Arp2/3 and is involved in Arp2/3 mediated actin polymerization *in vitro *, although less efficient than cortactin [43]. Second, HS1 binds to F-actin with its 37-amino-acid repeat domain [36], however, it contains only three and one-half repeat in contrast to cortactin. Third, also HS1-splice variants have been detected such as a variant lacking the 3^rd ^repeat of the F-actin binding domain in a systemic lupus erythematosus (SLE) patient resulting in increased apoptosis after B-cell receptor (BCR) stimulation [44]. Fourth, HS1 is sequentially phosphorylated on three tyrosine residues by various Src family tyrosine kinases [31,45] and two serine/threonine residues [30], although at different residues than cortactin [25]. Finally, both cortactin and HS1 can accumulate into podosomes, structures found in osteoclasts [46] and marcrophages [47], but also in RSV transformed cells [48] and carcinoma cells [49].

Although cortactin and HS1 share a high amino acid sequence and structural similarity, their functions differ considerable. In this paper, we compare their genomic organization in order to provide more insight into their evolution, which may form the basis towards understanding specific functions of both genes. We describe the genomic organization and the exon-intron boundaries for human cortactin. Both the genomic cDNA and deduced amino acid sequences of human cortactin were compared to cortactin and HS1 genes from other species. Genomic comparisons revealed the evolution and underscore the significance of the conserved F-actin binding repeat domain for HS1 and cortactin and the importance of alternative splicing for cortactin function.

## Results and discussion

### The genomic organization of cortactin homologues

**Figure 1:**
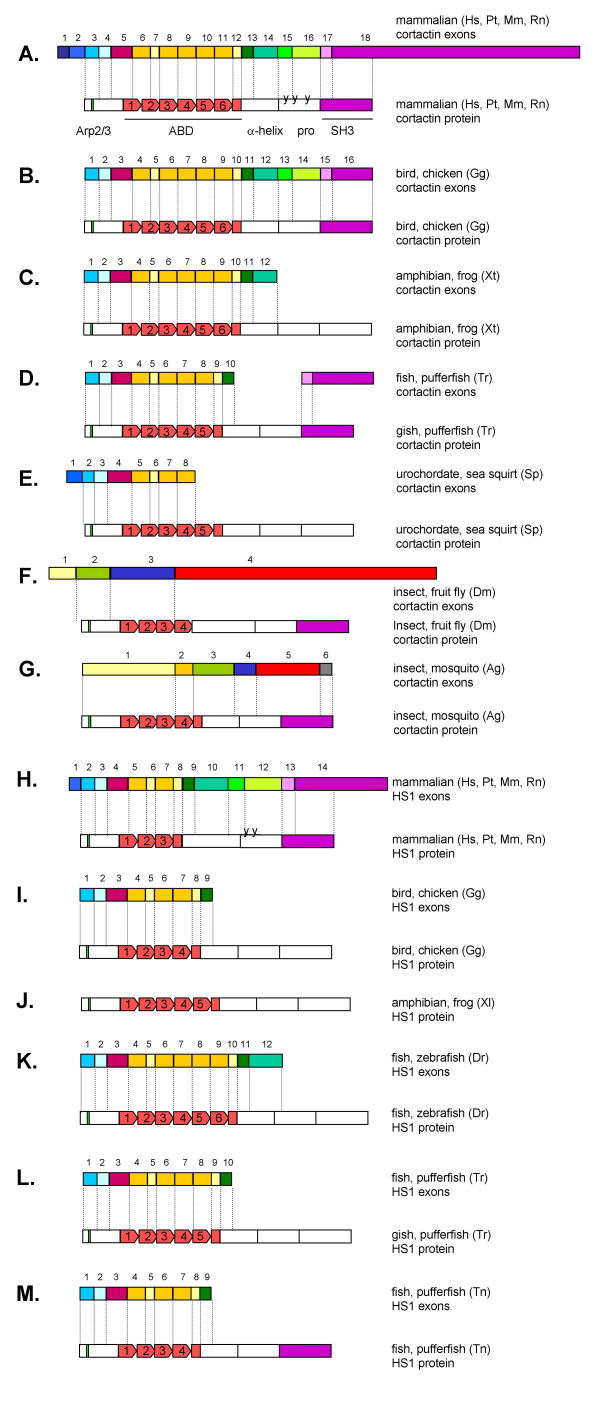
**Exon map of cortactin and HS1 from different species**. Exon/intron boundaries found in the genomic databases by performing BLAST searches with the cortactin cDNA of different species to their genomic DNA, are indicated as vertical boxes in different colors. A lack of boxes means that the boundaries were not found. The genomic organization of some species could not be fully elucidated, because cDNA/genomic sequences were not completely available. The actin binding repeat domain of the cortactin protein is represented by red boxes and the SH3 domain by the purple box. The vertical green stripe indicates the sequence coding for the Arp2/3 binding domain. Pro = proline rich region. The y in the proline rich region represents tyrosine phosphorylation sites. Hs, human; Pt, chimpanzee; Mm, mouse; Rn, rat.

We have previously described the isolation and sequencing of the *EMS1 *cDNA [28,49] (DDBJ/EMBL/GenBank Accession No. M98343) coding for the human cortactin protein. To evaluate the genomic structure, we determined the exon/intron-boundaries. Nucleotide sequence comparisons with human *EMS1 *cDNA sequence revealed homology with two human genomic clones (DDBJ/EMBL/GenBank Accession No. AP000487 and AP000405) (Table 1). The genomic structure of the *EMS1*/cortactin gene was determined by performing BLASTn comparisons of *EMS1 *cDNA against the genomic clones (Figure 1A). By amplifying the intron sequences (smaller than 2 Kb) using primers on adjacent exons followed by end-sequencing of these products, we confirmed the intron/exon boundaries of the human *EMS1*/cortactin gene. The *EMS1 *gene contains 18 exons spanning over about 38 Kb of genomic DNA. The length of the individual exons ranges from 55 to 178 bp, except the last exon (1564 bp). The splice donor and acceptor sequences, the sizes of the introns and exons of the human *EMS1/ *cortactin gene are provided in the supplementary materials [see Additional file 1]. The ATG is at position 169, at the first nucleotide of exon 3, indicating that the first two exons encode the 5' untranslated region (UTR). The F-actin-binding repeat domain is encoded by exon 5 to exon 12 with 5 exons of 111 nucleotides in length (exons 6, 8, 9, 10 and 11) (Figure 1A and [see Additional file 1]). The sequence encoding the DDW Arp2/3 binding site is located within exon 3 and the SH3 domain is encoded by exon 17 and 18. The 3' UTR is 1420 nucleotides in length with a polyadenylation signal AATAAA at position 3225.

**Table 1:** Accession numbers of cortactin and HS1 sequences

Gene	mRNA/EST	Protein	Genomic DNA	Chromosome
				
				
**COMPLETE CORTACTIN AND HS1 SEQUENCES**				
Human *(Homo sapiens, Hs)*				
*wt-cortactin*	M98343^a^	AAA58455	AP000487	11q13^b^
			AP000405	
*SV1-cortactin *^c^	BC008799	AAH08799		
	BC033889	AAH33889		
	NM_138565	NP_612632		
*HS1 *^d^	X16663	CAA34651	NT_005612	3q13
	BC016758	AAH16758		
				
Chimpanzee *(Pan troglodytes, Pt)*				
*wt-cortactin*			AADA01305241^e^	9
*HS1*			AADA01307895^e^	2
				
Mouse *(Mus musculus, Mm)*				
*wt-cortactin*	U03184	AAA19689	NT_00336 7F5	
*SV1-cortactin *^f^	BC011434	AAH11434		
	XM_144788	XP_144788		
	AK084249	BAC39148		
*HS1*	X84797	CAA59265	NW_006107	16B
	BC007469	AAH07469		
	D42120			
Rat *(Rattus Norvegicus, Rn)*				
*wt-cortactin*			NW_043405	*1q41*
*SV1-cortactin (isoform B)*	AF054619	AAC08425		
*SV2-cortactin (isoform C)*	AF054618	AAC08424		
*HS1*	XM_221421	XP_221421	NW_042728	11q11
				
Chicken *(Gallus gallus, Gg), wt-cortactin*	M73705	AAA49031	AADN01110316^g^	5
*SV1-cortactin*	BU109838^g^			
*HS1*		ENSGALG00000009778^e,p^		Un
				
Frog *(Xenopus laevis, Xl), wt-cortactin*	AB027611^h^	BAB79435		
Frog *(Xenopus tropicalis, Xt), wt-cortactin*			scaffold_32906^I^	
				
Zebrafish *(Danio rerio, Dr), wt-cortactin*	AF527956^i^	AAQ09010		
*HS1*			Finished_845^o ^	4
				
Pufferfish *(Takifugu rubripes, Tr), wt-cortactin*	SINFRUG00000156355^e^		scaffold_853^e^	
*HS1*	SINFRUG00000124755^e^		scaffold_1329^e^	
				
Pufferfish *(Tetraodon nigroviridis, Tn), HS1*		CAG04186	scaf14731	19
				
Fruit fly *(Drosophila melanogaster, Dm)*	NM_079702	NP_524426	AE003733	3R
	AB009998	BAA34397		
	AB030177	BAB01490		
				
Mosquito *(Anopheles gambiae, Ag)*	XM_315193	XP_315193	AAAB01008952^i^	2R
				
Sea urchin *(Strongylocentrotus purpuratus, Sp)*	NM_214617	NP_999782	scaffold_101^e^	
	AF064260^i^	AAD08655		
				
Sponge *(Suberites domuncula, Sd)*	Y18027	CAC38778		
	Y18860	CAC80140		
				
**INCOMPLETE CORTACTIN AND HS1 SEQUENCES**				
				
Cattle *(Bos taurus, Bt), SV1-cortactin*	TC154749^j,k^			
	B222447^k^			
Pig *(Sus scrofa), wt-cortactin*	TC48123^j,l^			
Frog *(Xenopus laevis, Xl) HS1*	BC060434	AAH60434		
Sea squirt *(Ciona intestinalis, Ci)*	TC32922^j,m^		scaffold_101^i^	
White shrimp *(Litopenaeus setiferus, Ls)*	BE846976			
White shrimp *(Litopenaeus vannamei, Lv)*	BE188605			
Root knot worm *(Meloidogyne incognita, Mi)*	BE188583			
	BQ613692^n^			
	BQ625292^n^			
Root knot worm *(Meloidogyne chitwood, Mc)*	CB856307			
	BQ613692			
Root knot worm *(Meloidogyne javanica, Mj)*	BE578389			

^a^All accession numbers except as noted below, may be found in the mRNA, EST, protein of genomic databases of NCBI [65].

^b^Chromosomal locations were obtained from UniGene, NCBI [65].

^c^SV, splice variant. Accession numbers from EST's BE714795, BE717740, BE717751, BE717765, BE717811, E717819, BE717829, BE717871, BE274120, BE728099 [65].

^d^HS1= Haematopoietic lineage cell-specific protein 1 = HCLS1= hematopoietic cell-specific Lyn substrate 1

^e^Accession number was obtained from the EnsEMBL [67].

^f^SV, splice variant. Accession numbers from EST's BE290787, BF321856, BG519413, BG174188, AA762862, AI099054, BF135250 [65].

^g^Accession numbers were obtained from the U.S. Poultry Gene Mapping Project [75].

^h^From 2500 bp untill 3578 bp of this mRNA is mRNA from another gene. Genomic DNA are pieces of sequences.

^i^Accession numbers were obtained from the DNA Data Bank of Japan [70].

^j^Accession numbers from EST's from the TIGR) [69].

^k^Homologue to actin binding domain of human SV1-cortactin.

^l^Homologue to C-terminal part of human cortactin incuding the SH3 domain.

^m^Homologue to repeat 1 to 5 of the actin binding domain of human cortactin.

^n^Accession numbers were obtained from the European Bioinformatics Institute. Homologue from 5' untills repeat 3 of the actin binding domain of human cortactin [73].

^o^The deduced cDNA and protein sequence from the genomic zebrafish Finished_845 sequence is more related to human HS1, while the zebrafish mRNA/protein sequence (AF527956, AAQ09010) showed more homology to human cortactin.

^p^The deduced cDNA and protein sequence from the genomic chicken ENSGALG00000009778 showed more homology to human HS1[67].

Other cortactin homologues have been reported in mouse [3], rat [19], chicken [50], fruit fly (*Drosophila melanogaster *) [51], and frog (*Xenopus laevis *) [20]. We searched in numerous databases for all known cortactin genes in other species (listed in Table 1). The identification is based on overall amino acid sequence and overall structural homology with human cortactin. Cortactin homologues exist in mammalians (human, chimpanzee, cattle, pig, mouse, rat), birds (chicken), amphibians (frog), fishes (zebrafish, pufferfish), urochordates (sea squirt), invertebrates (sea urchin), insects (fruit fly, mosquito), shrimps, worms and sponges. To date, there is no evidence for the existence of cortactin in unicellular species, nor in plants. Thus, cortactin seems to be restricted to metazoans.

For several species, both cDNA and genomic sequences (total or partial) are available and therefore we were able to reveal their genomic organization using BLASTn. The exon/intron-boundaries were determined and compared to human cortactin [see Additional file 1]. As schematically presented in Figure 1, the genomic organization and the lengths of the exons as well as the locations of the exon/intron boundaries are highly conserved from urochordates to mammalians. Pufferfishes have the shortest known genome of all vertebrate species due to much shorter introns, nevertheless most exon/intron boundaries were conserved and similar to mammalian cortactin. Intriguingly, the number of repeats in the actin-binding domain differs between species (Figure 1A–G). The number of exons and the location of the intron/exon borders of insect cortactin (*Drosophila *and mosquito) differ considerably with mammalian cortactin, despite the proteins sequences are very similar. *Drosophila *and mosquito carry 4 repeats in the actin-binding domain. In both species, repeat 1-to-3 and 4 are on separate exons with in mosquito the 4^th ^repeat of the actin binding domain to be encoded by a single 111 bp large exon 2 (Figure 1F,G). Both, sponge (the lowest metazoan) and sea squirt (urochordate) cortactin protein carry 5 repeats. During evolution, after creation of sponges and worms, the coelomata divided into insects and urochordates (that evolved later into vertebrates). The genomic organization of ancestors of the coelomata should reveal the roots of cortactin evolution. However, complete cDNA and/or genomic DNA of cortactin homologues in these species are not yet available.

### The genomic organization HS1 homologues

Both nucleotide and amino acid sequence comparisons with cortactin revealed the highest similarity with the hematopoietic lineage cell-specific protein 1 (HS1). So far, HS1 homologues have been reported in human [27], mouse [33], rat and chimpanzee (NCBI database), suggesting that HS1 exists in mammalians only. We determined the intron/exon boundaries of mammalian HS1 genes by aligning the cDNA with the genomic DNA using BLASTn (Figure 1H and [see Additional file 2]). The number and lengths of the exons and the locations of the exon/intron boundaries were very similar to cortactin, especially in the exons that encode the actin-binding domain (compare [see Additional file 1] and [see Additional file 2]). The exons 10–13 of HS1 encoding the centre region between the actin-binding domain and the SH3 domain are longer (633 bp versus 489bp in cortactin) and more divergent compared to corresponding exons of cortactin.

In addition to a single cortactin homologue in all other species, nucleotide sequences comparisons using the mammalian HS1 mRNA and genomic DNA sequences revealed (incomplete) genomic sequences in chicken, pufferfish, zebrafish and frog (Table 1 and Figure 1I–M) that were more related to the HS1 protein (Figure 3 and [see Additional file 3]). Because no HS1 homologues for these species were present in the mRNA/dbEST database (except for *X. laevis *HS1), the cDNA (and corresponding protein) sequences were deduced from the genomic DNA with BLASTn or were predicted by Ensemble program. In these lower species, two cortactin related proteins exist. To distinguish between cortactin and HS1 variants, only the most conserved N-terminal part of cortactin and HS1 protein variants, including repeat 3 (corresponding to amino acid 1–190 of human cortactin) was used in BLASTp analysis. In each species, one protein variant turned out to be more homologous to human cortactin, and was called cortactin, whereas the other protein variant appeared to be more related to HS1 and was called HS1. This analysis unveiled HS1 proteins with more than 3 repeats in chicken and pufferfish *Tetraodon nigroviridis *(containing 4 1/2 repeats), pufferfish *Takifugi rubripes *and *Xenopus laevis *HS1 (5 1/2 repeats) and zebrafish HS1 (6 1/2 repeats) (Figure 1 I-M).

Moreover, alignments of the exon/intron boundaries of these HS1 genes to the mammalian HS1 genes [see Additional file 2] revealed that exon 7 (repeat 3) of HS1 was most similar to exon 10 (repeat 5) of cortactin suggesting that in mammalians exon 8 and 9 (repeat 3 and 4) of HS1 were lost during evolution. This is supported by the presence of at least one sequence of 111 nucleotides in the 5670 bp intron 6 of human HS1 (location 3271–3381) that is predicted by the program HMMER when performing alignments using a consensus sequence of the 37 amino acid repeats. However, this sequence is not functional because it does not represent an exon based on the consensus sequence of exon-intron junctions ('gt ... ag' rule of intron sequences) and no human transcripts or ESTs of HS1 including this sequence are present in the NCBI databases. In summary, HS1 is not restricted to mammalians only, but exist also in fishes, amphibians and birds and its genomic structure is very similar to that of cortactin.

### Different promoter regions explain distinct tissue specificity of cortactin and HS1

**Figure 2:**
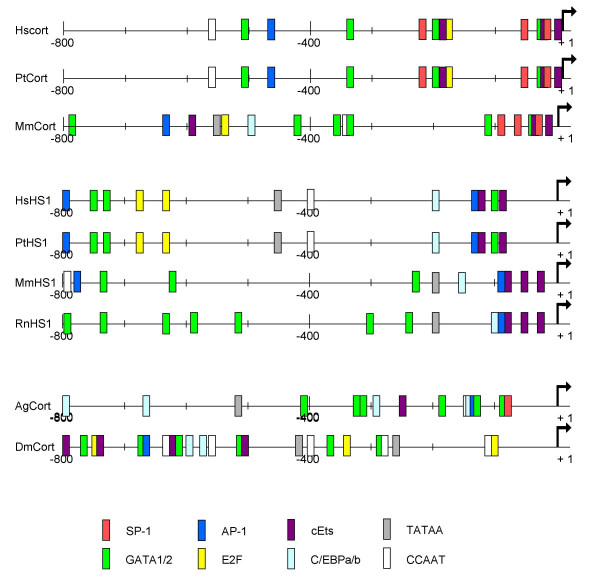
**A schematic view over 800 bp of the proximal promoters**. Distribution of putative binding sites where represented for the transcription factors SP1 (red), GATA1 or GATA2 (green), AP-1 (dark blue), E2F (yellow), cEts (purple), C/EBPa or C/EBPb (light blue) and the TATAA box (gray) and CCAAT box (white) in the promoter regions of cortactin, human (HsCort), chimpanzee (PtCort), mouse (MmCort), mosquito (AgCort), Drosophila (Dmcort), and HS1, human (HsHS1), chimpanzee (PtHS1), mouse (MmHS1) and rat (RnHS1). The mRNA starting point (assigned +1) is indicated by an arrow.

Cortactin is widely expressed in most cell types suggesting to be important for vital functions, while HS1 expression is restricted to hematopoietic cells suggesting to be tailored later in evolution to serve a specific function in these cells. In concordance with their tissue-specific expression pattern, we suppose that their expression might be differently regulated. Therefore, we compared the upstream promoter regions of several cortactin and HS1 genes (Figure 2). The mammalian cortactin gene is very GC rich and contains putative SP-1 transcriptional factor binding sites that are common to many TATA-less promoters and typical for promoter regions in 'widely-expressed housekeeping genes'. Ets family transcription factors, found in the HS1 promoters, are specific for hematopoietic cells and involved in controlling the expression of many B cell- and macrophage-specific genes [52] and are critical for development of lymphoid and myeloid cell lineages. The promoter region of *Drosophila *and mosquito cortactin shares putative transcription factors found both in mammalian cortactin and HS1. Thus at least in mammalians, the nature of the promoters seemed to determine the broad distribution of cortactin expression in various tissues except most hematopoietic cells and the limited expression of HS1 to hematopoetic cells.

**Figure 3:**
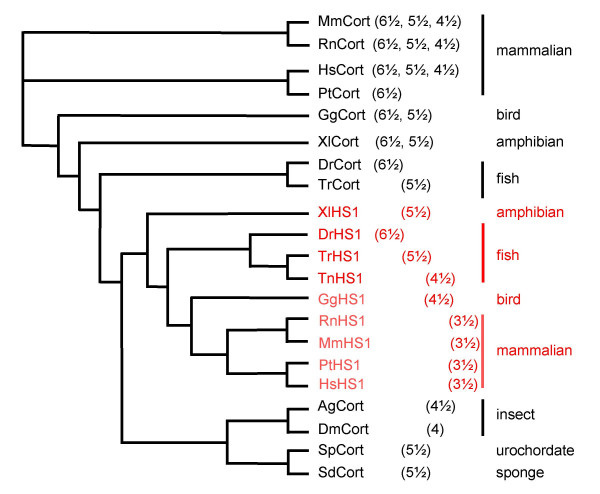
**Phylogenetic relationship of cortactin and HS1 genes**. Evolutionary comparison of the N-terminal of cortactin and HS1 proteins including repeat 3 (corresponding to nucleotide 1–190 of human cortactin), represented in a phylogenetic tree based on a cluster alogorithmic alignment generated using GeneBee ClustalW 1.83 program. The number of repeats in the full length actin binding domain for the indicated species are depicted between brackets. Hs, human; Pt, chimpanzee; Mm, mouse; Rn, rat; Gg, chicken; Xl, frog *Xenopus laevis *; Dr, zebrafish; Tr, pufferfish *Takifugu rubripes *; Tn, pufferfish *Tetraodon nigroviridis *; Dm, fruit fly *Drosophila *; Ag, mosquito; Sp, sea urchin; Sd, sponge.

### The significance of the actin binding repeat domain in cortactin and HS1

We recently reported the identification of two alternative splice variants of human cortactin; SV1-cortactin lacking the 6^th ^repeat and SV2 lacking the 5^th ^and 6^th ^repeat resulting in a different F-actin binding properties and decreased cell migration [14]. As shown in Table 1, cortactin splice variants exist in other mammalians as well as in chicken and frog. So far, splice variants in other species have not been identified, suggesting that alternative splicing of cortactin seems to be restricted to higher metazoans. All intron sequences of cortactin bordering the splice site junctions follow the general GT/AG rule [53] except for intron 11 (GC/AG) [see Additional file 1]. As has been shown for other genes, a GT-to-GC transition might be responsible for the generation of an alternatively mRNA transcript [54]. However, in frog (*Xenopus laevis *), the SV1-cortactin variant exists despite the splice donor of intron 11 begins with a GT [20]. Thus, concerning the genome of these different species, alternative splicing of the actin-binding domain of cortactin seems to be facilitated during evolution by modulating the splicing machinery by a GT-to-GC transition to create cortactin related variants that influences cellular properties [14]. The relative expression of cortactin splice variants by tissue origin [14] suggested that splice variants might have tissue-specific functions such as fine-tuning the organization of the F-actin cytoskeleton and consequently regulating cell adhesion and migration.

Alternative splicing also occurs in human HS1. Recently a splice variant lacking the 3^rd ^repeat (exon 7) has been found in an SLE patient [44], resulting in enhanced BCR-mediated cell death. This alternative splicing event was due to a germ line mutation. In contrast, the splice donor of HS1 intron 6 begins with a GC [see Additional file 2]. With respect to the similarities between cortactin and HS1, it might be of interest to investigate the occurrence of splicing of HS1 exon 6 and possible biological consequences. The 3^rd ^repeat and its NLS links HS1 to a role in apoptosis, while such a role has not been described for cortactin lacking a NLS. Since the cytoskeleton architecture in hematopoietic lineage cells is very different from that in adherent cells, it is likely that HS1 plays an important role in the construction of tissue-type specific actin networks. Other types of actin cytoskeleton factors, such as the Arp2/3 complex activators of the WASP family have been reported to have distinct tissue specific expression profiles as well. Thus, the apparent role of HS1 in apoptosis is likely due to its actin remodeling related function. Additionally, our genomic comparisons revealed that the 3^rd ^repeat of HS1 corresponds with the 5^th ^repeat of cortactin, and therefore it might be of interest to investigate whether cortactin SV2 variant (lacking the 5^th ^and 6^th ^repeat) might be involved in apoptosis.

The 4^th ^repeat of cortactin has been suggested to be required for F-actin-binding [17]. Genomic comparisons revealed that HS1 lacks this 4^th ^repeat. Nonetheless, HS1 does bind to F-actin and activate the Arp2/3 complex, although at a lower efficiency than cortactin [43]. This suggests that not only a single repeat but the number of repeats is crucial for the F-actin-binding affinity [14,18]. In addition, HS1 contains a PIP_2 _binding site in each of its 3 repeats, whereas cortactin has only one in the 4^th ^repeat. PIP_2 _reduces F-actin cross-linking by cortactin, probably due to competition for the same binding site. Due to its higher affinity for PIP_2 _[36], HS1 restores this cortactin/F-actin cross-linking process by trapping PIP_2_. This might be of importance in platelets and megakaryocytes where both, cortactin and HS1 are expressed. Taken together, the composition of the repeat domain is also involved in diverting the functions of both genes.

An elegant way to study the function of a protein is to perform loss-of-function experiments. So far, cortactin knock-out models have not yet been generated successfully, because deletion of one allele of cortactin leads to premature differentiation of embryonic stem cells (personal communication in [55]). However, complete loss-of-function mutants of the *Drosophila *cortactin gene were viable and fertile, except impaired border cell migration during oogenesis [56]. Down-regulation of cortactin by RNA interference, revealed an essential role for cortactin in dendritic spine morphogenesis [57] and in E-cadherin mediated contact formation in epithelial cells [58]. Mice lacking HS1, showed normal development of the lymphoid system [38], however, the antigen-receptor induced clonal expansion and deletion of B and T lymphocytes were impaired. Thus, loss of function studies underscores the divergent functions of HS1 and cortactin in different cell systems.

### Cortactin and HS1 are derived from an ancestral vertebrate cortactin-gene by gene duplication

To examine the genesis of the cortactin family, we studied the relationship between the cortactin and HS1 homologues by generating a phylogenetic tree based on a multi-sequence alignment with the ClustalW 1,83 program [see Additional file 3]. We compared the N-terminal regions including repeat 3 (corresponding to nucleotide 1 to 190 of human cortactin), because this is the best-conserved region among all homologues (Figure 3). One cluster contains all known HS1 proteins and appeared to be closest related to a cluster composed by insects (Mosquito (Ag), *Drosophila *(Dm)), urochordate (sea urchin, (Sp)) and sponge (Sd) cortactin. In this last cluster all the species with only one gene (with the highest similarity with cortactin) are present. This suggests that with the appearance of the vertebrates, an ancestral gene became duplicated to create two genes, which later evolved into cortactin and HS1. This hypothesis is supported by the fact that many genes duplicated at this stage in the evolution, the overall amino-acid sequence in both genes is very similar and the introns are located at the same amino acid position. Furthermore, gene duplication often correlates with a tissue specific expression pattern of the duplicated genes, which is true for mammalian cortactin and HS1.

**Figure 4:**
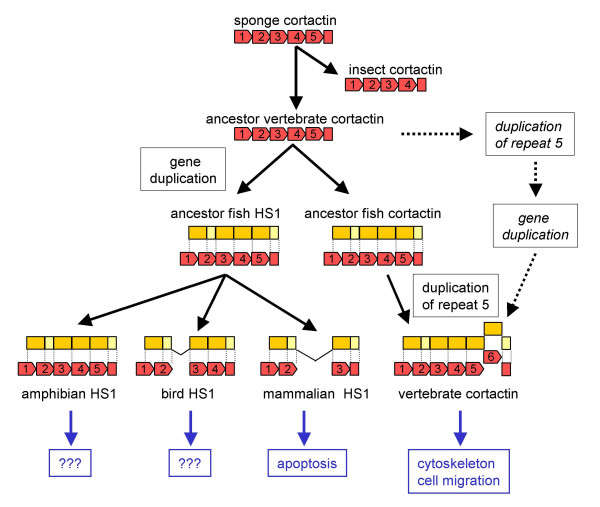
**Model for the origin of cortactin and HS1 during evolution**. Exon/intron boundaries from the exons encoding the actin binding repeat domain are represented in yellow. The actin binding repeat domain of the cortactin protein is represented by red boxes.

Figure 4 displays a hypothetical model for the origin of the cortactin and HS1 genes during evolution. The oldest ancestor is the sponge that, like sea squirt (urochordate), carries one cortactin protein with 5 c1/2 repeats. Insects have also one cortactin gene and evolved to 4 1/2 repeats. During evolution, after the creation of the sponge and the worms, the coelomata divided into insects and urochordates (that evolved later into vertebrates). This suggests that during the evolution, the number of repeats decreased in the insects. Unfortunately, no genomic sequences of ancestors of the coelomata that could reveal the roots of cortactin evolution are available yet to perform more detailed genomic analysis.

The genome of pufferfish *Takifugu rubripes *contains two cortactin-related genomic sequences both including 5 1/2 repeats. Most likely, an ancestor vertebrate cortactin gene underwent gene duplication. From this moment on during evolution, two cortactin/HS1-releated genes are present in all higher species. One gene evolved to mammalian HS1 with a specific function in apoptosis in hematopoietic cells. For its function, exon 8 and 9 (encoding repeat 3 and 4) were not useful and lost during evolution. However, the HS1 protein in pufferfish *Takifugu rubripes *and frog *Xenopus laevis *contains 5 1/2 repeats, while chicken and pufferfish *Tetraodon nigroviridis *HS1 carries 4 1/2 repeats. It might be of interest to investigate the function of these HS1 proteins and their functional differences to mammalian HS1. The other gene evolved to a ubiquitously expressed mammalian cortactin protein with a vital function in the organization of the cytoskeleton and cell migration. The 6^th ^repeat of cortactin most likely originated from a duplication event of the 5^th ^repeat, since the 6^th ^repeat is most similar to the 5^th ^repeat in all species with 6 1/2 repeats. We recently demonstrated that 6 1/2 repeats are necessary for optimal F-actin cross-linking activity and cell migration, while the splice variant lacking both the 5th and 6th repeats (SV2) was less efficient [14]. Thus, the number of repeats in the F-actin binding domain of cortactin fine-tunes its function in cytoskeletal remodeling. For that reason, in higher metazoans, alternative splicing of the F-actin binding domain is most likely facilitated by a GT-GC transition in the splice donor. Alternatively, we can not exclude that gene duplication might have taken place after duplicated of the 5^th ^repeat (dotted arrows), since both zebrafish cortactin and HS1 contain 6 1/2 repeats.

## Conclusions

We report the genomic organization of cortactin and HS1 genes of several species. These genes display a conserved genomic organization as the coding regions have almost identical exon/intron structure. Comparison of 5' sequences allows possible regulatory elements that stress their specific tissue distribution. Comparative analysis of the genomic organization and amino acid sequences of cortactin and HS1 provides insight into the evolution of the conserved actin-binding repeat domain, which forms the basis towards understanding specific functions of both genes. Most likely, both genes originated from a gene duplication event and subsequently HS1 lost two repeats, whereas cortactin gained one repeat. Our analysis genetically underscores the significance of the F-actin binding domain in cytoskeletal remodeling, which is of importance for the major role of HS1 in apoptosis and for cortactin in cell migration.

## Methods

### The genomic structure of human cortactin

To determine the genomic structure of the human cortactin gene, an algorithm was applied based on the consensus sequence of exon-intron junctions ('gt ... ag' rule of intronic sequence) as well as on the codon usage within ORF. Nucleotide sequence comparisons with human cortactin sequences (NCBI, GenBank accession no. M98343) using BLASTn [59] revealed homology with two genomic clones (GenBank accession no. AP000487 and AP000405). With these clones, we determined all exon/intron boundaries and size of all introns and exons (Table 2A) of the human cortactin gene by (1) performing BLAST comparisons with the cDNA against the genomic DNA and (2) using the GeneFinder program [60] based on the consensus sequence of exon-intron junctions ('gt ... ag' rule of intronic sequence) as well as on the codon usage within ORF [61].

To confirm the predicted genomic structure, we determined the intron/exon boundaries using a cloning procedure as described [62]. Genomic DNA of two cosmid clones COS-7.12 and COS-3.72 covering the cortactin gene as determined by the full-length cDNA [5], was amplified with randomly selected primers from the cDNA sequence (GeneBank accession no. M98343). All PCR products that were larger than the cDNA control sample were considered to be caused by intron sequences and compared to genomic sequence (accession number AP000487 and AP000405) using BLASTn [59]. The size of intron 1, 5, 8, 12 and 13 was too large to obtain a reliable sequence.

Because no overlapping genomic sequences immediately 5' of the first exon were present in the database, we performed sequence analysis of a 2.7-kb HincII-HincII fragment representing the first exon and its 5'-flanking sequences from cosmid COS-7.12 cloned into pUC18 (p5'EMS_3135). In addition, we sequenced a 5-kb PCR product using a 5'-primer in the vector (within the TET gene) and 3'-primer (p3135p601: 5'-ccgggtcggccctggattcc-3') within exon 1, subcloned in pUC18 (p5'EMS_4911). Nucleotide sequences of both products were compared with the genomic clones representing the cortactin gene present in the NCBI database (Accession number AP000487 (GI 8118774 and GI 6277297) and AP000405 (GI 8118742)) and used to define the 7.4 kb 5'-flanking region. The PROSCAN program [63] from BIMAS was used to define the 316 bp promoter region preceding exon 1. Putative transcription factor binding sites where determined by the TFSEARCH program [64] and graphically represented in figure 2. Sequences from human cortactin were submitted to NCBI GenBank [65] as accession No. M98343 (cDNA) and AJ288897 (promoter).

### Database searching

The (deduced) protein and genomic sequences of all cortactin and HS1 genes were retrieved from various WEB-sites and their available sequence data are summarized in Table 1. In addition, partial cortactin sequences (ESTs and/or genomic) of various organisms were identified based on amino acid sequence homology with existing cortactin proteins. The genomic organization of the sea squirt and Takifugu rubripes could not be completely elucidated, because cDNA/genomic sequences were only partially available. All data were compiled using BLAST searches of the following databases: National Center for Biotechnology Information (NCBI) (Bethesda, MD, USA) [65]; The Wellcome Trust Sanger Institute (Cambridge, UK) [66]; EnsEMBL of The Wellcome Trust Sanger Institute (Cambridge, UK) [67]; DOE Joint Genome Institute (Walnut Creek, CA, USA) [68]; TIGR: The Institute for Genomic Research (Rockville, MD, USA) [69]; DNA Data Bank of Japan (Mishima, Shizuoka, Japan) [70]; Nematode.net Genome Sequencing Center (St. Louis, MO, USA) [71]; Wormbase (NY, USA) [72]; European Bioinformatics Institute (EBI) (Cambridge, UK) [73]; Genoscope National Sequencing Center (Evry, France) [74]; The U.S. Poultry Gene Mapping Project (MI, USA) [75] and UCSC Genome Bioinformatics (Santa Cruz, CA, USA) [76].

To determine the exon/intron boundaries of all cortactin and HS1 genes, available genomic sequences were subjected to sequence alignments of each species-specific cDNA sequence using the BLAST program of NCBI. Using the same algorithms, as described for human cortactin, the exon/intron-boundaries could be predicted. The complete genomic sequences of the 5' flanking region of cortactin of human, chimpanzee, mouse, rat, fruit fly, and mosquito were determined using the various accession numbers of genomic DNA in Table 1. Putative transcription factor binding sites of 800 bp of the 5' flanking regions where determined by the TFSEARCH program (Figure 2). The predicted exon in intron 6 of HS1 was predicted by the bio-informatics program HMMER [77]) The human cortactin 6 1/2 repeats of the actin-binding domain were aligned, resulting in a consensus sequence: (kfGvqkdrvDksAvGfdyqekvekhesqkDysk). With HMMER this consensus sequence was 'tBLASTn' to intron 6 of human HS1. With an acceptable probability (E-value 0.095), the program predicted an exon in this intron 6 (at location 3271–3381).

### Amino acid sequence comparisons

Sequence alignments were carried out using the BLAST program of NCBI. The multiple sequence alignments of various cortactin proteins were constructed using Basic GeneBee ClustalW 1.83 [78]. The genome, cDNA or protein was completed for all cortactin homologues and the number of repeats differs across species and between HS1 and cortactin. Only the N-terminal of cortactin and HS1 proteins including repeat 3 (corresponding to amino acid 1–190 of human cortactin) was used to generate a phylogenetic tree, because this is the most conserved part. Predicted nuclear localization signals sequences were obtained using Predict NLS program [79].

## List of abbreviations

aa, amino acid(s); bp, base pair(s); BCR, B-cell receptor; EST, expressed sequence tag; HS1, hematopoietic lineage cell-specific protein 1; NLS, nuclear localization signal; RT-PCR, reverse transcriptase polymerase chain reaction; SH3, Src homology; UTR, untranslated region.

## Authors' contributions

AGSHvR designed the study on comparative genome analysis, performed database searches, sequence alignments and gene structure prediction and drafted the manuscript. ESS designed, conducted and analyzed the cloning and sequencing of the promoter of human cortactin. VvBvS conducted and analyzed the PCR and sequencing experiments of the exon-intron boundaries of human cortactin and its splice variants. PMK read the manuscript and provided comments. ES helped with writing the paper, provided overall technical guidance and coordination. All authors read and approved the final manuscript.

## Supplementary Material

Additional File 1 Splice donor and acceptor sequences of cortactin in different species. Click here for file

Additional File 2 Splice donor and acceptor sequences of HS1 in different species. Click here for file

Additional File 3 Multiple amino acid sequence alignment of cortactin and HS1 homologues. Click here for file
