# Supplementary material for: Comparative genome analysis of cortactin and HS1: the significance of the F-actin binding repeat domain
Source: BMC Genomics. 2005 Feb 14;6:15. doi: 10.1186/1471-2164-6-15 (PMC554100; doi:10.1186/1471-2164-6-15)
Supplement: Additional File 1 — Splice donor and acceptor sequences of cortactin in different species. [file 1471-2164-6-15-S1.doc]

**Splice donor and acceptor sequences of cortactin in different species.** Adjacent exon (uppercase letters) and intron (lowercase letters) sequences are given for each junction (p, purine residue; q, pyrimidine residue; n, any residue). Underlined letters in the splice consensus sequences indicate residues that are inflexible in the consensus sequence. The underlined ATG and TAG represent the start and stop codon respectively. Eukaryotic introns are categorized as type 0, 1 or 2 introns, depending on their phase, i.e. the position of their 5’-boundary within a triplet of the protein-coding sequence. Type 0 introns interrupt the reading frame between two triplets, while the boundary of type 1 introns is located after the first nucleotide of a triplet. The introns of the actin-binding domain of cortactin are all of type 1 (intron 7-11), indicating that splicing in this region will not lead to truncated proteins. *, exon-intron boundaries of Xenopus laevis were previously published [20]. Chimp, chimpanzee; chick, chicken; zebra, zebrafish; puff, pufferfish.

______________________________________________________________________________________

Sequences at exon-intron junction of cortactin gene ______________________________________________________________________________________________

Exon Size 5’splice donor Size Intron 3’splice acceptor

No. exon intron type

(bp) (bp)

1 71 GCGGCG gtgagc 6650 0 taatctttttacag ACGGAA human (Hs)

71 GCGGCG gtgagc 6809 taatctttttacag ACGGAA chimp (Pt)

40 TTGGCG gtgcgg 4727 tcttctcttcatag GTAATC mouse (Mm)

2 97 AGAAAG gtactt 1936 0 ggctgctctttcag ***ATG***TGG human (Hs)

97 AGAAAG gtactt 1931 ggctgctctttcag ***ATG***TGG chimp (Pt)

94 AGAAAG gtttgt 1955 tgccgctcttgcag ***ATG***TGG mouse (Mm)

tgctgcccttgcag ***ATG***TGG rat (Rn)

tggtggtgttgcag ***ATG***TGG chick (Gg)

tttatttcttccag ***ATG***TGG frog (Xt)

***ATG***TGG puff (Tr)

3 87 TTTGTG gtagga 133 0 gcttttcttttcag AATGAT human (Hs)

87 TTTGTG gtagga 133 gcttttcttttcag AATGAT chimp (Pt)

87 TTTGTG gtagga 535 tcttctctttgcag AATGAT mouse (Mm)

87 TTTGTG gtagga 520 acttctctttgcag AACGAT rat (Rn)

84 TTTGTG gtatgg 435 tctgtccttcgcag AATGAC chick (Gg)

81 TTTGTG gtaagt 1026 ttttttttggacag AATGAT frog (Xt)

84 TTTGAG gtacct 85 cttttttcatctag AATGAT puff (Tr)

4 74 TATCAA gtaaga 2239 2 tctcttccatcaag CATACA human (Hs)

74 TATCAA gtaaga 2239 tctcttccatcaag CATACA chimp (Pt)

74 CATCAA gtaagt 1994 ttcctcccctccag CATTCA mouse (Mm)

74 CATCAA gtaagt 2024 tttctccactctag CATACA rat (Rn)

74 CATCAA gtaaag 1320 gtctgtctttaaag TATTCA chick (Gg)

74 CATTAA gtatgt 617 cctgttcttttcag TATCCA frog (Xt)

74 CATCAA gtgagg 81 cgtgtccggcccag TATCCA puff (Tr)

5 130 GATAAG gtaagt 4581 0 gaaccctgttccag TCAGCT human (Hs)

130 GATAAG gtaagt 5137 gaaccctgttccag TCAGCT chimp (Pt)

130 GACAGA gtaagt 3399 tattctttgtccag TCAGCC mouse (Mm)

130 GACAAA gtaagt 3883 cttctttgttccag TCAGCT rat (Rn)

130 GATAAA gtaagt 2393 gcactttcttttag TCAGCT chick (Gg)

130 GATAAG gtgcgt 1519 ccttctctgcctag ATGGCT frog (Xt)

130 GACAAG gtttag 695 tgtgtgttattcag TCTGCA puff (Tr)

6 111 GATCAG gtgagt 1010 0 tttggtcgtcacag TCTGCT human (Hs)

111 GATCAG gtgagt 1021 tttggttgtcacag TCTGCT chimp (Pt)

111 GATCAG gtgcgt 1173 tttccgtcccacag TCTGCT mouse (Mm)

111 GATCAG gtgagt 1289 tttttgtcccacag TCTGCT rat (Rn)

111 GACCAG gtaagc 681 tatttctcttccag TCAGCT chick (Gg)

111 GATCAG gtaagc 484 catttctcttgcag TCTGCT frog (Xt)

111 GATCAG gtattt 293 ctgtctcattccag TCTGCT puff (Tr)

7 55 AGAAAG gtaaga 1295 1 ccctgtctctccag ACTACT human (Hs)

55 AGAAAG gtaaga 1360 ccctgtctctccag ACTACT chimp (Pt)

55 AGAAAG gtaggg 1655 tcttgtctccttag ACTACT mouse (Mm)

55 AGAAAG gtaagg 1551 tcttgtctccttag ACTACT rat (Rn)

55 AAAAAG gtaaag 515 tcctctcaatgcag ACTACT chick (Gg)

55 AGAAAG gtgagc 1229 cttgcgtctgacag ATTATA frog (Xt)

55 AGAAAG gtaacg 363 gctttgctgtgcag ATTACT puff (Tr)

8 111 AGAGAG gtgggg 2622 1 ttgtttgtttttag ATTACT human (Hs)

111 AGAGAG gtgggg 2630 ttgtttgtttttag ATTACT chimp (Pt)

111 AGAAAG gttcgg 2016 tgttgttgtttcag ATTACT mouse (Mm)

111 AGAAAG gttcat 1549 tgttcttgtttcag ATTACT rat (Rn)

111 AAAAAG gtgagc 795 actcttatctctag ATTATT chick (Gg)

111 AGAAAG gtttgt >431 frog (Xt)

111 AGAAAG gttggt 267 ctgttcaccggcag ATTACA puff (Tr)

9 111 AGAAAG gtgtct 543 1 cattgtgcatgtag ACTATG human (Hs)

111 AGAAAG gtgtct 543 cattgtgcatgtag ACTATG chimp (Pt)

111 AGAAAG gtacct 410 tgttgtgcatgcag ACTATG mouse (Mm)

111 AGAAAG gtacct 422 tgttgtgcatgcag ACTATG rat (Rn)

111 AGAAAG gttttt 576 cttcacgcatacag ATTATG chick (Gg)

taatttgctcatag ATTATT frog (Xl)

111 AGAAAG gtcggt 320 ggtgtgtttctcag ACTACG puff (Tr)

10 111 AAAAAG gtacat 959 1 ttgtggattttcag ATTATA human (Hs)

111 AAAAAG gtacat 960 ttgtggattttcag ATTATA chimp (Pt)

111 AAAAAG gtacct 938 ccgtggatctccag ACTATA mouse (Mm)

111 AAAAAG gtacct 756 acgtggatctccag ACTATA rat (Rn)

111 AGAAAG gtatat 1247 tgtggctttttcag ACTATA chick (Gg)

111 AGAAAG gttcag ±2900 ttctatgtgtgtag ACTATG frog (Xl)*

111 AGAAAG gtgaag 127 puff (Tr)

11 111 AGCAAG ***gc***acag 1341 1 ttgccacgtttcag ACTACT human (Hs)

111 AGCAAG ***gc***acag 1377 ttgccacgtttcag ACTACT chimp (Pt)

111 AGCAAG ***gc***acag 1362 ttaccaccttttag ACTATG mouse (Mm)

111 AGCAAG ***gc***acag 1414 ttaccaccttttag ACTATG rat (Rn)

111 AACAAG ***gc***accg 1696 gtcctgctttttag ATTATT chick (Gg)

111 AAAAAG gtacac ±2100 gcctcttatttcag ATTATT frog (Xl)*

tttttccttaacag ATTACT puff (Tr)

12 56 GATAAG gtaaat 2336 0 tcctgtctctgcag AATGCG human (Hs)

56 GATAAG gtaaat ? tcctgtctctgcag AATGCG chimp (Pt)

56 GACAAG gtaaga 3686 ctctgtctccatag AATGCA mouse (Mm)

56 GACAAG gtaagg 3694 ctctgtctccacag AACGCA rat (Rn)

56 GATAAG gtaagt 376 ttctttaacaatag AATGCA chick (Gg)

56 GATAAG ccccatgtttccag GCTGCA frog (Xl)*

56 GACAAG gtgagg 156 tctctgatcaacag ACGGCT puff (Tr)

13 70 AAGCTG gtgagt 3649 1 tccttcctctatag TGACCA human (Hs)

70 AAGCTG gtgagt ? TGACCA chimp (Pt)

70 AGGCCG gtgagt 3955 tctctttcctctag TAACCA mouse (Mm)

70 AGGCTG gtgagt 3806 tctctttcctctag TGACCA rat (Rn)

70 AAGCTG gtaaga 499 tcttaatgttacag TCGCTA chick (Gg)

70 AAGTCG gtaggt tgtttgccccacag AGGGCA frog (Xl)

70 AGGTGT gtcagt puff (Tr)

14 149 CTGGAG gtgagt 1991 0 gttttcaatcacag GAGCAA human (Hs)

149 CTGGAG ? gttttcaatcacag GAGCAA chimp (Pt)

149 CTGGAA gtgagt 1600 ctgtcctgtcttag GAGCAA mouse (Mm)

149 CTGGAA gtgagt 1885 tccctttgtcttag GAGCAA rat (Rn)

149 CTGGAG gt*c*agt 9666 tctcttcatttaag GAGCAA chick (Gg)

149 CAGGAG gttggt frog (Xl)

15 90 TATGAG gttggt 1820 0 tgacccttccccag GATGCG human (Hs)

90 TATGAG gttggt 1820 tgacccttccccag GATGCG chimp (Pt)

90 TATGAG gtgagt 1087 gtgtccctccctag GATGCA mouse (Mm)

90 TATGAG gtgggt 1026 gtatctttcctcag GATGCA rat (Rn)

90 TACCAG gtttgt 87 aattgtttttgcag GATGCA chick (Gg)

16 178 CCGCAG gtactg 368 1 tggctttcttttag AGGACA human (Hs)

178 CCGCAG gtactg 368 tggctttcttttag AGGACA chimp (Pt)

166 AAGCAG gtactg 745 ctgcttttttacag AGGATG mouse (Mm)

166 AAGCAG gtactg 1665 ctgctttcttacag AGGATG rat (Rn)

190 AAGCAG gtaagg 642 tctctctccctcag AGGAAA chick (Gg)

17 72 AGGCTG gtgagc 1307 1 gttctcttccccag CGGGCG human (Hs)

72 AGGCTG gtgagc 1305 gttctcttccccag CGGGCG chimp (Pt)

72 AGGCTG gtaagg 847 ctctccttccctag CTGGCG mouse (Mm)

72 AGGCTG gtaaga 794 ctctccctccctag CTGGCG rat (Rn)

72 AGGCTG gtaagt 794 tgctctctctccag CGGGTG chick (Gg)

18 1564 1819 ***TAG****(stop codon) human (Hs)

1537 1819 ***TAG****(stop codon) chimp (Pt)

1368 1769 ***TAG****(stop codon) mouse (Mm)

1527 1524 ***TAG****(stop codon) rat (Rn)

491 1689 ***TAG****(stop codon) chick (Gg)

Splice concensus sequences

AG gtpagt qqqqqqqqqqncag GT
