# Supplementary material for: Comparative genome analysis of cortactin and HS1: the significance of the F-actin binding repeat domain
Source: BMC Genomics. 2005 Feb 14;6:15. doi: 10.1186/1471-2164-6-15 (PMC554100; doi:10.1186/1471-2164-6-15)
Supplement: Additional File 2 — Splice donor and acceptor sequences of HS1 in different species. [file 1471-2164-6-15-S2.doc]

**Splice donor and acceptor sequences of HS1 in different species.** Adjacent exon (uppercase letters) and intron (lowercase letters) sequences are given for each junction (p, purine residue; q, pyrimidine residue; n, any residue). Underlined letters in the splice consensus sequences indicate residues that are inflexible in the consensus sequence. The underlined ATG and TAG represent the start and stop codon respectively. Eukaryotic introns are categorized as type 0, 1 or 2 introns, depending on their phase, i.e. the position of their 5’-boundary within a triplet of the protein-coding sequence. Type 0 introns interrupt the reading frame between two triplets, while the boundary of type 1 introns is located after the first nucleotide of a triplet. The introns of the actin-binding domain of cortactin are all of type 1 (intron 7-11), indicating that splicing in this region will not lead to truncated proteins. *, exon-intron boundaries of Xenopus laevis were previously published [20]. Chimp, chimpanzee; chick, chicken; zebra, zebrafish; puff, pufferfish. The exon numbers between brackets correspond with analogous exons of cortactin in Additional file 1.

______________________________________________________________________________________

Sequences at exon-intron junction of HS1 gene ___________________________________________________________________________________________

Exon Size 5’splice donor Size Intron 3’splice acceptor

No. exon intron type

(bp) (bp)

1 (2) 72 GTGGAG gtgggt 2472 0 tctcag ***ATG***TGG human (Hs)

49 GTGGAG gtgggt 2452 tctcag ***ATG***TGG chimp (Pt)

60 GTAAAG gtgggt 2212 cctcag ***ATG***TGG mouse (Mm)

62 GGAAAG gtgggt 2223 cctcag ***ATG***TGG rat (Rn)

ctgcag ***ATG***TGG chick (Gg)

ttatag ***ATG***TGG zebra (Dr)

tgccag ***ATG***TGG puff (Tr)

ttccag ***ATG***TGG puff (Tn)

2 (3) 84 TTTGTG gtgagt 911 0 ttgcag AATGAC human (Hs)

84 TTTGTG gtgagt 907 ttgcag AATGAC chimp (Pt)

84 TTTGTG gtgagt 462 ttgtag AATGAC mouse (Mm)

84 TTTGTG gtaagt 515 ttgtag AATGAC rat (Rn)

84 TTTGTG gtgagt 1253 ctgcag AACGAC chick (Gg)

84 TTTGAG gtaaga 105 gtttag AATGAT zebra (Dr)

84 TTTGAG gtactt 67 tttcag AATGAC puff (Tr)

84 TTTGAG gtaccc 68 tttcag AACGAC puff (Tn)

3 (4) 74 CATCAA gtaggt 9830 2 ttccag CATCCA human (Hs)

74 CATCAA gtaggt 9795 ttccag CATCCA chimp (Pt)

74 CATCAA gtaggt 8687 ttgcag CATCCA mouse (Mm)

74 TATCAA gtaggt 8390 ttgcag CATCCA rat (Rn)

74 CATCAA gtgagt 128 ctgcag CATCCA chick (Gg)

74 CATCAG gtaact 421 gtgtag CATTGC zebra (Dr)

74 CATCAG gtagga 295 caacag TGTGGC puff (Tr)

74 CATCAG gtgggt 263 cgacag TGTGGC puff (Tn)

4 (5) 130 GACAAG gtaagt 2390 0 tctcag AGTGCA human (Hs)

130 GACAAG gtaagt 2034 tctcag AGTGCA chimp (Pt)

130 GACAAG gtgagt 1830 ccttag AGTGCC mouse (Mm)

130 GACAAG gtgagt 1700 cctcag AGTGCC rat (Rn)

130 GACAAG gtaaca 865 cctcag TGTGCC chick (Gg)

130 GACAAG gttaga 1455 tcccag GGTGCT zebra (Dr)

124 GACAAG gtttat 83 atctag GTAGCT puff (Tr)

124 GACAAG gtttgt 66 gtccag GTGGGC puff (Tn)

5 (6) 111 GACAAG gtaagt 1836 0 ctgtag TCAGCA human (Hs)

111 GACAAG gtaagt 1836 ctgtag TCAGCA chimp (Pt)

111 GACAAG gtaggt 2468 tctcag TCAGCG mouse (Mm)

111 GACAAG gtaggt 2628 tctcag TCAGCG rat (Rn)

111 GACAAG gtgcac 583 ttccag TCGGCG chick (Gg)

111 GACAAG gttaga 139 ctccag TCTGCC zebra (Dr)

111 GACAAG gtggga 88 ttccag TCCGCC puff (Tr)

111 GACAAG gtggga 66 ctccag TCCGCC puff (Tn)

6 (7) 55 AGAAAG ***gc***aagg 5670 1 ccacag ATTACT human (Hs)

55 AGAAAG ***gc***aagg 5677 ccacag ATTACT chimp (Pt)

55 AGAAAG ***gc***aagt 4102 tctcag ATTACT mouse (Mm)

55 AGAAAG gtaagt 2878 tttcag ATTACT rat (Rn)

55 AGAAAG ***gc***aagt 1591 cctcag ATTACT chick (Gg)

55 AGAAAG gtaagt 84 cctaag ATTACG zebra (Dr)

55 AGAAAG gttatc 78 ctttag ATTATT puff (Tr)

55 AGAGAG gtggag 68 ctttag ATTATT puff (Tn)

(8) 111 AGAAAG gtgagc 134 ctgaag ACTACT chick (Gg)

111 AGAAAG gtctct 92 tcgcag ACTACG zebra (Dr)

111 AGAAAG gtggga 64 ccgcag ACTACG puff (Tr)

111 AGAGGG gtgagc 64 tgccag ACTACT puff (Tn)

(9) 111 AGAAAG gtaagt 1004 tcacag ACTACG zebra (Dr)

111 AGAAAG gtgagt 70 gctcag ATTACT puff (Tr)

7 (10) 111 AGAGAG gtgagt 656 1 ctgcag ATTATG human (Hs)

111 AGAGAG gtgagt 655 ctgcag ATTATG chimp (Pt)

111 AGAGAG gtaaat 593 ccacag ATTATG mouse (Mm)

111 AGCGAG gtaagt 609 ctacag ATTATG rat (Rn)

111 AAACAG gtacag 290 atacag ACTATG chick (Gg)

111 AGAAAG gtaagt 170 ttacag ACTATG zebra (Dr)

111 AGAAAG gtgagg 67 gttcag ATTACT puff (Tr)

111 AGAGAG gtgagg 71 gtccag ATTACA puff (Tn)

(11) 111 AGAAAG gtattg 128 cggtag ACTATG zebra (Dr)

8 (12) 56 GATAAG gtaaag 629 0 ctgcag AGCGCT human (Hs)

56 GATAAG gtaaag 629 ctgcag AGCGCT chimp (Pt)

56 GATAAG gtaaac 551 ttgcag AGTGCT mouse (Mm)

56 GATAAG gtaagc 561 ttgcag AGTGCT rat (Rn)

56 GATAAG gtgagt 493 ttgcag AGTGCT chick (Gg)

56 GATAAG gtaaac 979 ccacag AATGCA zebra (Dr)

56 GATAAG gtcagt 239 atctag AGCGCA puff (Tr)

56 GACAAG gtcagc 159 ctgtag AGCGCA puff (Tn)

9 (13) 70 AAGCCG gtgagg 1316 1 ctgcag CTTCTA human (Hs)

70 AAGCCG gtgagg 1327 ctgcag CTTCTA chimp (Pt)

70 AAGCTG gttagt 571 ctgcag CTTCCA mouse (Mm)

70 AAGCTG gttagt 616 ctgcag CTTCCA rat (Rn)

70 AAGCAG gtgaga 210 catcag CTTCCA chick (Gg)

70 AGGGCT gtgagt zebra (Dr)

70 AGGCCT gtatgt puff (Tr)

70 AGGCCT gtacgt puff (Tn)

10 (14) 212 TCAGAG gtgagc 1035 0 ccacag GCCTGG human (Hs)

212 TCAGAG gtgaga 1037 ccacag GCCTGG chimp (Pt)

212 TCAGAG gtgcgt 4011 cttcag GTCTGC mouse (Mm)

212 TCAGAG gtgagt 484 cttcag GTCTGG rat (Rn)

11 (15) 105 CCGGAG gtaagc 503 0 tctcag GACAAT human (Hs)

105 CCGGAG gtaagc 502 tctcag GACAAT chimp (Pt)

117 TTGGAG gtaagc 440 cctcag GACAAC mouse (Mm)

117 CTCGAG gtgagc 440 cctcag GACAAT rat

12 (16) 232 TGGCTG gtgagt 147 1 atgcag GATCAT human (Hs)

232 TGGCTG gtgagt 147 atgcag GATCAT chimp (Pt)

244 AAGCTG gtgagt ? attcag GACCCT mouse (Mm)

214 AGGCTG gtgagt 130 acccag GACACT rat (Rn)

13 (17) 84 AAGGAG gtaggt 118 1 ccccag AGGGAA human (Hs)

78 AAGGAG gtaggt 120 ccccag AGGGAA chimp (Pt)

63 AAGGAG gtaagt 185 ccccag AGGGAA mouse (Mm)

63 AAGGAG gtaagt 185 ccccag AGGGAA rat (Rn)

14 (18) 583 1501 ***TAG****(stop codon) human (Hs)

585 1501 ***TAG****(stop codon chimp (Pt)

597 1481 ***TAG****(stop codon) mouse (Mm)

597 1491 ***TAG****(stop codon) rat (Rn)

Splice concensus sequences

AG gtpagt qqncag GT
