# Supplementary material for: Comparative genome analysis of cortactin and HS1: the significance of the F-actin binding repeat domain
Source: BMC Genomics. 2005 Feb 14;6:15. doi: 10.1186/1471-2164-6-15 (PMC554100; doi:10.1186/1471-2164-6-15)
Supplement: Additional File 3 — Multiple amino acid sequence alignment of cortactin and HS1 homologues. [file 1471-2164-6-15-S3.doc]

**Multiple amino acid sequence alignment of cortactin and HS1 homologues.** Multiple amino acid sequence alignment was constructed using GeneBee CLUSTAL W (1.83) program [78]. "*" = identical or conserved residues in all sequences in the alignment; ":" = indicates conserved substitutions; "." = indicates semi-conserved substitutions. Predicted nuclear localization signals (NLS) sequences were obtained using Predict NLS program [79]. The putative PIP_2_ binding (consensus R/K YG V/I E/D R/K D R/K) [36], DDW-Arp2/3 binding sites [16] and phosphorylation sites (yellow) were previously revealed in human and mouse and extrapolated to other species. The conserved 37 aa repeat domain are indicated in green alternating in blue, the last repeat of 23 aa is indicated in purple. The SH3 domain is indicated in blue letters. HsCort, human; PtCort, chimpanzee; MmCort, mouse; RnCort, rat; GgCort, chicken; XlCort, frog *Xenopus laevis*; DrCort, zebrafish; TrCort, pufferfish *Takifugu rubripes*; TnCort, pufferfish *Tetraodon* *nigroviridis*; DmCor, fruit fly *Drosophila melanogaster*; AgCort, mosquito; SpCort, urochordate sea urchin; SdCort, sponge.

TrHS1 ----------MWRSAVGHSVDMK-VSA-EGDDWETDPDFENDVSEQEQRWGAKTIEGSG-

TnHS1 ----------MWRSAVGHNVEVK-VSK-EGDDWETDPDFENDVSEQEQRWGAKTIQGSG-

HsHS1 ----------MWKSVVGHDVSVS-VET-QGDDWDTDPDFVNDISEKEQRWGAKTIEGSG-

PtHS1 ----------MWKSVVGHDVSVS-VET-QGDDWDTDPDFVNDISEKEQRWGAKTIEGSG-

MmHS1 ----------MWKSVVGHDVSVS-VET-QGDDWDTDPDFVNDISEKEQRWGAKTIEGSG-

RnHS1 ----------MWKSVVGHNVSVS-VET-QGDDWDTDPDFVNDISEKEQRWGAKTIEGSG-

HsCORT ----------MWKASAGHAVSIA-QDDAGADDWETDPDFVNDVSEKEQRWGAKTVQGSG-

PtCORT ----------MWKASAGHAVSIA-QDDAGADDWETDPDFVNDVSEKEQRWGAKTVQGSG-

MmCORT ----------MWKASAGHAVSIT-QDDGGADDWETDPDFVNDVSEKEQRWGAKTVQGSG-

RnCORT ----------MWKASAGHAVSIT-QDDGEADDWETDPDFVNDVSEKEQRWGAKTVQGSG-

GgCORT MTVLLLVVLQMWKATAGHSIAVS-QDD-GADDWETDPDFVNDVSEKEQRWGAKTVKGSG-

XlCORT ----------MWKSAAGHSLSIS-TDE--TDDWETDPDFVNDIDEKEQRWGAKTVTGSG-

DrCORT ----------MWKAAAGQSVSMS-VDEG-ADDWETDPDFENDVSEKEQRWGAKTVQGSG-

DmCORT ----------MWKASAGHQIQATSAASAEDDDWETDPDFVNDVSEQEQRWGSKTIDGSGR

AgCORT ----------MWKSTAGRDIDTTGVNQGEDDDWETDPDFVNDVSEQEQRWGSKTVEGSGR

SpCort ----------MWRSQVGSSNKPP-VTQDEDDDWETDPDFVNDVSEQEQRWGSKSVEGSG-

SdCORT ----------MWRAQLGSKEVKT-ITQADDDDWETDPDFVNDVTEEEQRWGSKTVDGSVE

**:: * ***:***** **: *:*****:*:: **

PIP_2_

TrHS1 RKEHISVAELRKKVAVEHEQVKQK--DQTP-KASYGYGGKFGVEKDRMDKVAVGTNYVAQ

TnHS1 RKEHIRVAELRKEVAVEHEQVKQK--DQTP-KASYGYGGKFGVEKDRMDKVALGNDYVAS

HsHS1 RTEHINIHQLRNKVSEEHDVLRKKEMESGP-KASHGYGGRFGVERDRMDKSAVGHEYVAE

PtHS1 RTEHINIHQLRNKVSEEHDVLKKKEMESGP-KASHGYGGRFGVERDRMDKSAVGHEYVAE

MmHS1 RTEHINIHQLRNKVSEEHDILKKKELESGP-KASHGYGGQFGVERDRMDKSAVGHEYVAD

RnHS1 RTEHINIHQLRNKVSEEHDILKKRELESGP-KASHGYGGRFGVERDRMDKSAVGHEYVAE

HsCort HQEHINIHKLRENVFQEHQTLKEKELETGP-KASHGYGGKFGVEQDRMDKSAVGHEYQSK

PtCort HQEHINIHKLRENVFQEHQTLKEKELETGP-KASHGYGGKFGVEQDRMDKSAVGHEYQSK

MmCort HQEHINIHKLRENVFQEHQTLKEKELETGP-KASHGYGGKFGVEQDRMDRSAVGHEYQSK

RnCort HQEHINIHKLRENVFQEHQTLKENELETGP-KASHGYGGKFGVEQDRMDKSAVGHEYQSK

GgCort HQEHINIHQLRENVFQEHQTIKEKELETGP-KASHGYGGKFGVEQDRMDKSAVGHEYQSK

XlCort HQEHINIHQLRQNVSHEHKEIKEKELEVGP-KASHGYGGKFGVEKDRMDRSAVGHEYQTK

DrCort HQEHINIHKLRQTVSTEHSDLKQKELATMP-KASHGYGGKFGLHQDRMDKSAVGHEYQSK

DmCort TAGTIDMDKLREETEQADLDKKKQLLKDQN--AGYGYGGKFGVEKDRMDKSAVGHDYQGK

AgCort NAAAIDMQQLREETERADSEKKR--KEGPK--ASHGYGGKFGVEKDRMDKSAVGHEHIEK

SpCort RQEQFNIHELRENVKQGDADQKEKELAAAP-KASYGYGGKFGVQQDRMDKSAVGHDHQES

SdCort RKGALSMSQIREDVKKEDTVVKAKTTHHSQSDSSKGFGGKYGVQKERQDKSAVGWDYQAN

: : ::*: . . : :. *:**::*:.::* *: *:* :: .

PIP_2_

TrHS1 VEKHSSQKDASKGFGGKFGVQEDRVDKVGTQFQYKGEVEQHTSQKDYSKGFGGKYGVEKE

TnHS1 VDKHSSQKDASKGFGGKFGVEKDRVDKSALGFGYKGEVEQHTSQRDYSKGFGGKYGVEKE

HsHS1 VEKHSSQTDAAKGFGGKYGVERDRADKSAVGFDYKGEVEKHTSQKD--------------

PtHS1 VEKHSSQTDAAKGFGGKYGVERDRADKSAVGFDYKGEVEKHTSQKD--------------

MmHS1 VEKHSSQTDAARGFGGKYGVERDRADKSAVGFDYKGEVEKHASQKD--------------

RnHS1 VEKHSSQTDAAKGFGGKYGVERDRADKSAVGFDYKGEVEKHASQKD--------------

HsCort LSKHCSQVDSVRGFGGKFGVQMDRVDQSAVGFEYQGKTEKHASQKDYSSGFGGKYGVQAD

PtCort LSKHCSQVDSVRGFGGKFGVQMDRVDQSAVGFEYQGKTEKHASQKDYSSGFGGKYGVQAD

MmCort LSKHCSQVDSVRGFGGKFGVQMDRVDQSAVGFEYQGKTEKHASQKDYSSGFGGKYGVQAD

RnCort LSKHCSQVDSVRGFGGKFGVQMDRVDQSAVGFEYQGKTEKHASQKDYSSGFGGKYGVQAD

GgCort LSKHCSQVDSVKGFGGKFGVQTDRVDQSAVGFEYQGKTEKHASQKDYSSGFGGKYGVQAD

XlCort LSKHCSQSDSAKGFGGKFGVQTDRVDQSAVNFDYKGKTEKHASQKDYATGFGGKYGVQAD

DrCort LSKHCSQTDTSKGCGGQYWLQDDRVDHSAVGFQYVGKTEKHXSKTDYSTVLGGRYGVHAD

DmCort VGKHASQKDYSDGFGGKFGVQEDRKDKSAVGWDHVEKVEKHASQKDYATGFGGKFGVQSD

AgCort VEKHASQKDYVSGFGGKFGVQKDRVDKSAHGWDHVEKVDKHESQKDYKTGFGGKFGVQQD

SpCort LNKHASQADYAKGFGGKHGVQSDRQDASAVGFDYEGKTDKPASQKDYSSGFGGKFGVQKQ

SdCort LAKHGSQTDAAKGFGGKYGVQDANKDKSAVGWDYQANLAKHGSQTDAAKGFGGKYGVTE-

: ** ** * * **:. :: . * . : : : : *: * :**::**

PIP_2_

TrHS1 KVDKSALGYDYKGQTEKHQSQKDYAKGFGGKYGVEKEKVDKAALGYDYKGQTEKHQSQKD

TnHS1 KVDKSALGYDYKGQTEKHQSQRD-------------------------------------

HsHS1 ------------------------------------------------------------

PtHS1 ------------------------------------------------------------

MmHS1 ------------------------------------------------------------

RnHS1 ------------------------------------------------------------

HsCort RVDKSAVGFDYQGKTEKHESQRDYSKGFGGKYGIDKDKVDKSAVGFEYQGKTEKHESQKD

PtCort RVDKSAVGFDYQGKTEKHESQRDYSKGFGGKYGIDKDKVDKSAVGFEYQGKTEKHESQKD

MmCort RVDKSAVGFDYQGKTEKHESQKDYSKGFGGKYGIDKDKVDKSAVGFEYQGKTEKHESQKD

RnCort RVDKSAVGFDYQGKTEKHESQKDYSKGFGGKYGIDKDKVDKSAVGFEYQGKTEKHESQKD

GgCort RVDKSAVGFDYQGKTEKHESQKDYSKGFGGKYGVDKDKVDKSAVGFEYQGKTEKHESQKD

XlCort RVDKSALGFDYKGKTEKHESQKDYTTGFGGKFGVQADRVDKSAVGYDYQGKTEKHESQKD

DrCort RVDHVAVGFDYQGKTEKHESQKDYSKGFGGKYGIDKDKVDKSAVGFEYQGKTEKHESQKD

DmCort RVDKSAVGWDHIEKVEKHESQKD-------------------------------------

AgCort RQDKSAVGWDHIEAPQKHESQID-------------------------------------

SpCort SMDKSAVGWDYQAGLSQHNSQKDYSTGFGGKHGVQTDRQDASAVGFDYEGKTEKHASHKD

SdCort NKDKNAVGWDYQANLAKHESQTDAAKGFGGKYGVQTDSQDKNAAGWDYQEKLSQHSSQKD

*:

PIP_2_

TrHS1 YSKGFGGKFGVEREKVDKAALGYDYKSKTEKHQSQKDYSSGFGGRYGVQTDRMDKSAAGF

TnHS1 YSRGFGGKFGVEREKVDKAALGYDYKGETEKHQSQRDYTSGFGGRYGVQTDRMDKSAAGF

HsHS1 YSRGFGGRYGVEKDKWDKAALGYDYKGETEKHESQRDYAKGFGGQYGIQKDRVDKSAVGF

PtHS1 YSRGFGGRYGVEKDKWDKAALGYDYKGETEKHESQRDYAKGFGGQYGIQKDRVDKSAVGF

MmHS1 YSHGFGGRYGVEKDKRDKAALGYDYKGETEKHESQRDYAKGFGGQYGIQKDRVDKSAVGF

RnHS1 YSHGFGGRYGVEKDKRDKAALGYDYKGETEKHESQRDYAKGFGGQYGIQKDRVDKSAVGF

HsCort YVKGFGGKFGVQTDRQDKCALGWDHQEKLQLHESQKDYKTGFGGKFGVQSERQDSAAVGF

PtCort YVKGFGGKFGVQTDRQDKCALGWDHQEKLQLHESQKDYKTGFGGKFGVQSERQDSAAVGF

MmCort YVKGFGGKFGVQTDRQDKCALGWDHQEKLQLHESQKDYKTGFGGKFGVQSERQDSSAVGF

RnCort YVKGFGGKFGVQTDRQDKCALGWDHQEKLQLHESQKDYAKGFGGKFGVQSERQDSSAVGF

GgCort YVKGFGGKFGVQTDRQDKCALGWDHQEKVQLHESQKDYKSGFGGKFGVQTERQDPSAVGF

XlCort YSKGFGGKYGVDKENVDKSAVGFDYQGKTEKHESQKDYVKGFGGKFGVQTDRQDKCALGW

DrCort YVKGFGGKFGVQTDRQDKCALGWDHQEKLQLHESQKDYKTGFGGKFGVQSERQDSSAVGF

DmCort YSKGFGGKFGVQEDRKDKSAVGWDHKEAPQKHASQVD-----------------------

AgCort HKVGFGGKFGVQTDRKDKSAFGWDHVEKPQMHESQLDHKIGFGGKFGVQNDRMDKSAVGF

SpCort YSSGFGGKYGVQKDSQDSSAVGFDYEGKTEKHASQTDSSKGFGGKFGVDKNAQDATAGGF

SdCort GAKGFGGKYGVQSESQDKSALGYDHQTGLSKHGSQTDAAKGFGGKYGVEEGNQDSSAGGY

.*.*:*: . * ** *

NLS

TrHS1 SDMDSPTSAYEKTEPFEACMFLAQQDAGKLKARFESMAKASDEENRRKA-----------

TnHS1 SDMDAPTSSYEKTQPFEAS----SADAGKLKARFESMAKASGEENRRKA-----------

HsHS1 NEMEAPTTAYKKTTPIEAA----SSGARGLKAKFESMA----EEKRKRE-----------

PtHS1 NEMEAPTTAYKKTTPIEAA----SSGARGLKAKFESMA----EEKRKRE-----------

MmHS1 NEMEAPTTAYKKTTPIEAA----SSGARGLKAKFESLA----EEKRKRE-----------

RnHS1 NEMEAPTTAYKKTTPIEAA----SSGARGLKAKFESMA----EEKRKRE-----------

HsCort DYKEKLAKHESQQDYSKGF----GGKYGVQKDRMDKNASTFEDVTQVSS-----------

PtCort DYKEKLAKHESQQDYSKGF----GGKYGVQKDRMDKNASTFEDVTQVSS-----------

MmCort DYKERLAKHEPQQDYAKGF----GGKYGVQKDRMDKNASTFEEVVQVPS-----------

RnCort DYKERLAKHEPQQDYAKGF----GGKYGVQKDRMDKNASTFEEVVQVPS-----------

GgCort DYKEKLAKHESQQDYSKGF----GGKYGVQKDRMDKNAATFEDIEKPTS-----------

XlCort DHQEKLQLHESQKDYSQGF----GGKYGVQKDRMDKAAASFEDVEKVSS-----------

DrCort DYKERLAKHEPQQDYAKGF----GGKYGVQKDRMDKNASTFEEVVQVPS-----------

DmCort ----------HKVKPVIEG-----AKPSNLRAKFENLAKNSEEESRKRA-----------

AgCort QEQDKIGTNYTKVKPDIGS-----AKPSNLRAKFENFAATAEEEARKRA-----------

SpCort GDMQGVSSSYKKTRPQPPA----KSGAGNMRNKFEQMAQAGEEESRRKAEEERGRRQARE

SdCort DDMQAVKSDHRTERG--VS----KGQTGSIRSKFENMAVA--------------------

: :::. *

NLS

TrHS1 ----EEEKARRRARESREREVARHRQEVHAHN-TRWHVPDVVP-----------------

TnHS1 ----EEEKARRRARESRERELAERRQEVGARARRRQHAPAAFASAFHSFSCRRRGAEERT

HsHS1 ----EEEKAQQVARRQQERKAVTKRSPEAPQPVIAMEEPAVPAPLPKKISS------EAW

PtHS1 ----EEEKAQQVARRQQERKAVTKMSPEAPQPVIAMEEPAVPAPLPKKISS------EAW

MmHS1 ----EEEKAQQMARQQQERKAVVKMSREVQQPSMPVEEPAAPAQLPKKISS------EVW

RnHS1 ----EEEKAQQMARQQQERKAMVKMSREAQQPSVPVDEPAAPAPLPKKISS------EVW

HsCort ----AYQKTVPVEAVTSKTSNIRANFENLAKEKEQEDRRKAEAER---------------

PtCort ----AYQKTVPVEAVTSKTSNIRANFENLAKEKEQEDRRKAEAER---------------

MmCort ----AYQKTVPIEAVTSKTSNIRANFENLAKEREQEDRRKAEAER---------------

RnCort ----AYQKTVPIEAVTSKTSNIRANFESLAKEREQEDRRKAEAER---------------

GgCort ----TYQKTKPVERVANKTSSIRANLENLAKEKEQEDRRKAEAER---------------

XlCort ----SYQKTRPVEVEGSKASSIRANFENLAKDKEQEDRKKAEAER---------------

DrCort ----AYQKTVPIEAVTSKTSNIRANFENLAKEREQEDRRKAEAEP---------------

DmCort ----EEQKRLREAKDKRDREEAAKKTVAENTPRTSTEAPPPKGSRAAIQTG---RTGGIG

AgCort ----DEQKRLREEKDRCDREEAAKR---LNHSAESAEPKKPE-RKGPINTG---REAGVS

SpCort QKEKEADKKKEEERQRALAEHHRALPDEPEPEQEPEREPAPEPQRAR------------L

SdCort --------------------------------------EAPPPQ----------------

TrHS1 --EVDNPPPEVDNPPPEVDNPPPDVRANTPTI-------EERPE--------SEVKLGFT

TnHS1 SRRTSRRTSQTRRRSLSVHHPLSARRERMWKK-------EQSRR--------LHFLMHFL

HsHS1 PPVGTPPSSESEPVRTSREHPVPLLPIRQTLP----EDNEEPPA--------LPPRTLEG

PtHS1 PPVGTPPSSESEPVRTSREHPVPLLPIRQTLP----EDNEEPPA--------LPPRTLEG

MmHS1 PPAESHLPPESQPVRSRREYPVPSLPTRQSPLQNHLEDNEEPPA--------LPPRTPEG

RnHS1 PPAESHLPPQSEPVGGRREYPVPSLPTRQPPPQNPLEDNEEPPA--------LPPRTPEG

HsCort AQRMAKERQEQEEARRKLEEQAR-AKTQTPPVSPAPQPTEERLP--------SSPVYEDA

PtCort AQRMAKERQEQEEARRKLEEQAR-AKTQTPPASPAPQPTEERLP--------SSPVYEDA

MmCort AQRMAKERQEQEEARRKLEEQAR-AKKQTPPASPSPQPIEDRPP--------SSPIYEDA

RnCort AQRMAQERQEQE-ARRKLEEQAR-AKKQTPPASPSPQPAEDRPP--------SSPIYEDT

GgCort AQRMAREKQEQEEARRKLEEQAK-AKKQTPPPSPTTQPAEPKTP--------SSPVYQDA

XlCort AQRLERERREQEQARRQQEEQEN-AKPQTPPASP-----KATVP--------ESPIYEDA

DrCort TNTDSNTQSSPKPGRLNSPFLSKQSCDPEPFRSPVRQASPLRAA--------VASFEEQP

DmCort NAISAFNQMQSPVSETPPARKEPIIIPKAQPVKIEVEAKEEPTASTTSAAVAPTPTVVPA

AgCort SAISNFNNPQENITKEK-TRKDPIVLPK----------QDEPPK-------FVQPDVIPS

SpCort PEPAAAPRREPPPAAADDEWETQADQEEPPALPPPRQSKQLPSL--------PGRQAQPL

SdCort -APRKTPKYEPTVTEDEQTYPQQSYEEE--------QVEELYEV--------DQGRGE--

.

TrHS1 DIRRSITYFQMN----------------PQLSPG---------------FDKRNN-----

TnHS1 LTFQEEPQYDEP----------------PSLPPRSSDFLEAEAEADAPLLPNRSP-----

HsHS1 LQVEEEPVYEAE----------------PEPEPEPEP------EPENDYEDVEEM-----

PtHS1 LQVEEEPVYEAE----------------PEPEPEPEP------EPENDYEDVEEM-----

MmHS1 LQVVEEPVYEAA----------------PELEPEPEPDYEPEPETEPDYEDVGEL-----

RnHS1 LQVVEEPVYEAA----------------PEPEPEPEP----------DYEDVGEL-----

HsCort ASFKAELSYRGP-------------VSGTEPEPVYSM-------EAADYREAS-------

PtCort ASFKAELSYRGP-------------VSGTEPEPVYSM-------EAADYREAS-------

MmCort APFKAEPSYR-----------------GSEPEPEYSI-------EAAGIPEAG-------

RnCort APLKAEPSYG-----------------SSEPEPEYST-------EAAGLPEAS-------

GgCort VSYDAESAYKNSSTTY---------SAEHEPESGYKT-------TGSDYQEAV-------

XlCort SPVYESEAHR-------------------EP-PVSEP-------VYEDHQDAG-------

DrCort RSQYVEAEAE-----------------AEAEYEDVQV-------VSDKYEAPV-------

DmCort REPETAPVAKATAPPPDVVPQIEVETVDTPPRSEPQSPVYVPTPQPEVLAQVQVQP----

AgCort SDSTSVPTAKTKE--------------DQPEESAGHA---VQEPLPVARTSYSATP----

SpCort PSTPDEPNLYED---------------TGDVGTNFDE-------PEDTYADADATATYDQ

SdCort ----EEQVAYED---------------QPVDNEPVQE-------PEEAYQD---------

TrHS1 -----------QFN-VSLTDDA------EYEDLWR-------------------------

TnHS1 -----------QVDEEEEEDTG------VYEDLGELAPPAPADVKALICQL---------

HsHS1 -----------DRHEQEDEPEG------DYEEVLEPEDSSFSSALAGSSGCPAGAGAGA-

PtHS1 -----------DRHEQEDEPEG------DYEEVLEPEDSSFSSALAGSSGCPAGAGAG--

MmHS1 -----------DRQD--EDAEG------DYEDVLEPEDTPSLSYQAGPS-----AGAG--

RnHS1 -----------DRPD--EEAEG------DYEDVLEPEDTPSLSYQAGHS-----AGAG--

HsCort -----------SQQGLAYATEA------VYESAEAPGHYPAEDSTYDEYEN---------

PtCort -----------SQQGLAYATEA------VYESAEAPGHYPAEDSTYDEYEN---------

MmCort -----------SQQGLTYTSEP------VYETTEAPGHYQAEDDTYDGYES---------

RnCort -----------NQQGLAYTSEP------VYETTEVPGHYQAEDDTYDGYES---------

GgCort -----------SQREAEYEPET------VYEVAGAGDHYQAEENTYDEYEN---------

XlCort -----------GHQ--EAEQEA------VYESAD----YQEEENTYDEYSE---------

DrCort -----------EQQQPQYEAVE------EPDAVYENPTQVEEQSTYEPVD----------

DmCort -------EPQPQADPEPVVEEEPLYQNQAEIKAASPLPPTNGTVSEAVAPSGTATVPEEA

AgCort -------IAVEETDEAPVTDS------TAHQEQTTDTVPVQDDVEEFILSP---------

SpCort PPDDMGQQTYEEVDRAPVADDS------TYEDLPPQNEPVEDTTYEAVPEG---------

SdCort -----------EPEEPPAAPEE------VYQDEP---EPEEPTVVES-------------

SH3

TrHS1 ---------------GQTAVAIYDYVGEADDEISFNPDDIITHIEMIDEGWWKGQCR-GH

TnHS1 -------------SCGQRAKAIYDYVGEADDEISFNPEDIITHIEMIDEGWWRGECR-GR

HsHS1 ------------VALGISAVALYDYQGEGSDELSFDPDDVITDIEMVDEGWWRGRCH-GH

PtHS1 -------------ALGISAVALYDYQGEGSDELSFDPDDVITDIEMVDEGWWRGRCH-GH

MmHS1 -------------GAGISAIALYDYQGEGSDELSFDPDDIITDIEMVDEGWWRGQCR-GH

RnHS1 -------------GAGISAVALYDYQGEGSDELSFDPDDVITDIEMVDEGWWRGRCH-GH

HsCort -------------DLGYTAVALYDYQAAGDDEISFDPDDIITNIEMIDDGWWRGVCK-GR

PtCort -------------DLGYTAVALYDYQAAGDDEISFDPDDIITNIEMIDDGWWRGVCK-GR

MmCort -------------DLGITAIALYDYQAAGDDEISFDPDDIITNIEMIDDGWWRGVCK-GR

RnCort -------------DLGITAIALYDYQAAGDDEISFDPDDVITNIEMIDDGWWRGVCK-GR

GgCort -------------ELGITAIALYDYQAAGDDEISFDPDDIITNIEMIDDGWWRGVCK-GR

XlCort -------------DLGITAIALYDYQAAGDDEISFDPDDIITNIEMIDEGWWRGRCK-EC

DrCort -------------ERGVCARALYDYQAADDTEISFDPDDIITSIEMIDEGWWRGYGPDGH

DmCort IYANSDNLADYLEDTGIHAIALYDYQAADDDEISFDPDDVITHIEKIDDGWWRGLCK-NR

AgCort ------------DNPGIQAIALYDYQAAADDEISFDPDDKITHIEMIDEGWWRGWCN-NK

SpCort -------------QDGLRAKALYDYQATAEDELTFDPNEIITHVETIDDGWWKGVCR-GK

SdCort ---------------GLRAKAVYDYQATGEDEISFDPDDIIENIEQVDEGWWIGDFN-GN

* * *:*** . . *::*:*:: * :* :*:*** *

SH3

TrHS1 VGLFPAVYVKL-----

TnHS1 TGLFPALYVQLL----

HsHS1 FGLFPANYVKLLE---

PtHS1 FGLFPANYVKLLE---

MmHS1 FGLFPANYVKLL----

RnHS1 FGLFPANYVKLL----

HsCort YGLFPANYVELRQ---

PtCort YGLFPANYVELRQ---

MmCort YGLFPANYVELRQ---

RnCort YGLFPANYVELRQ---

GgCort YGLFPANYVELRQ---

XlCort CGLFPANYVELRQ---

DrCort FGMFPANYVELL----

DmCort YGLFPANYVQVVGQNS

AgCort YGLFPANYVQLLQ---

SpCort VGLFPANYVEMLYIR-

SdCort RGLFPANYVELI----

*:*** **::
